# Vemurafenib combined with chemotherapy achieved sustained remission in pediatric LCH: a multi-center observational study

**DOI:** 10.1007/s00432-023-05551-y

**Published:** 2024-01-17

**Authors:** Jiaying Lei, Wenxia Wang, Danna Lin, Chengguang Zhu, Wenguang Jia, Wenjun Weng, Xiaoshan Liu, Yuhan Ma, Zhixuan Wang, Lihua Yang, Xiangling He, Yunyan He, Yang LI

**Affiliations:** 1 Department of Pediatric Oncology, Sun Yat-Sen Memorial Hospital, Sun Yat-Sen University, Guangzhou, 510120 China; 2 Department of Pediatric Hematology and Critical Care Medicine, Zhujiang Hospital, Southern Medical University, Guangzhou, 510280 China; 3 Department of Pediatric Hematology and Oncology, Hunan Provincial People’s Hospital, Changsha, 410005 China; 4 The First Affiliated Hospital of Guangxi Medical University, Nanning, 530021 Guangxi China

**Keywords:** Langerhans Cell Histiocytosis, Vemurafenib, Chemotherapy, Children

## Abstract

**Background:**

Langerhans cell histiocytosis (LCH) is a myeloid neoplasia with potentially fatal consequences, and about 2/3 of cases involve the BRAF^V600E^ kinase-activated mutation. Vemurafenib, a BRAF inhibitor, has demonstrated significant clinical improvements in LCH. However, the high relapse rate of LCH following cessation of vemurafenib therapy remains a major challenge, and alternative treatment strategies require further investigation.

**Methods:**

In this retrospective multi-center study, we evaluated the efficacy and safety of vemurafenib combined with conventional chemotherapy in patients with severe or refractory LCH.

**Results:**

Seventeen patients were enrolled in the study, with eleven classified as risk organ involvement (RO +). Six received the combination therapy as the primary treatment, and eleven after being refractory to prior chemotherapy. The overall response rate was 94.1%. Progression-free survival among all 17 patients was 70.6% (12/17) at a median follow-up of 32 months, and relapse-free survival among the 15 patients with discontinuation after a response was 73.3%(11/15) at a median follow-up of 34 months. Five of six patients (83.3%) with myeloid BRAF^V600E^ mutations demonstrated molecular remission. The overall survival rate was 100%. Adverse events were mostly classified as grades 1 or 2.

**Conclusion:**

Our data suggest that the combination of vemurafenib and chemotherapy can achieve sustained clinical and molecular level relief in children with LCH, and side effects are tolerable.

**Supplementary Information:**

The online version contains supplementary material available at 10.1007/s00432-023-05551-y.

## Background

Langerhans cell histiocytosis (LCH) is an inflammatory neoplasia resulting from clonal expansions of CD207 + CD1a + cells, which causes tissue destruction and a wide range of organ involvement (Allen et al. 2018; Goyal et al. 2022). This disease predominantly affects children, and its clinical presentation can range from mild lesions to life-threatening conditions (Krooks et al. 2018). Patients with single lesions may respond positively to local treatment, while those with multisystem disease may be recurrent and refractory to systemic therapy (Cui et al. 2023). Despite the development in LCH treatment, approximately 30% of high-risk LCH patients experienced treatment failure, and 27% reactivate (Rodriguez-Galindo 2021; Tang et al. 2021; Sakamoto et al. 2023), increasing the risk of long-term complications and death. However, there is no ideal second-line therapy currently available. The treatment options include cladribine with cytarabine, MAPK pathway inhibitors (BRAF or MEK inhibitors), and hematopoietic stem cell transplantation. 2-CdA/Ara-C (cladribine and cytarabine) is an impressive salvage treatment but is associated with high toxicity (Donadieu et al. 2015). Furthermore, TNF-ɑ antagonist and hematopoietic stem cell transplantation are performed in patients refractory to standard chemotherapy (vinblastine, prednisone, and mercaptopurine); however, their dubious curative effects and adverse effects limit their widespread use (Rodríguez et al. 2009; Chohan et al. 2012; Akkari et al. 2003). The high toxicity and potential long-term adverse effects associated with salvage treatment highlight the need for innovative therapies in this patient group.

One promising targeted therapy for LCH is BRAF^V600E^ inhibitors since the genetic defect driving clonally expanded myeloid precursors to differentiate into LCH cells and cause lesions has been demonstrated to result from this mutation (Rollins 2015; Yang et al. 2021; Bigenwald et al. 2021). Vemurafenib is an oral low-molecular-weight molecule that selectively binds to the ATP-binding site of the kinase and inhibits BRAF^V600E^ (Garbe and Eigentler 2018). Previous studies have shown that vemurafenib generated nearly universal responses (Mohapatra et al. 2022); however, most patients experience rapid reactivations following therapy discontinuation. The persistence of BRAF^V600E^-positive cells in both blood and bone marrow suggests that vemurafenib can interrupt pathogenic mechanisms but is ineffective in eliminating LCH precursor cells (Eckstein et al. 2019). Therefore, alternative approaches are needed for a cure. Conventional chemotherapies can effectively destroy LCH cells and compensate for vemurafenib’s shortcomings. However, the available data regarding the efficacy of vemurafenib plus conventional chemotherapies in curing LCH are limited. We conducted a retrospective analysis to evaluate the safety and effectiveness of this treatment regimen in patients with severe or refractory LCH.

## Methods

### Patients

This was a multi-center retrospective study that involved LCH patients aged between 0 and 14 years receiving vemurafenib plus chemotherapies at four hospitals between January 2019 and December 2021, namely Sun Yat-sen Memorial Hospital (Guangzhou, China), People’s Hospital of Hunan Province (Hunan, China), Zhujiang Hospital of Southern Medical University (Guangzhou, China), and The First Affiliated Hospital of Guangxi Medical University (Guangxi, China). Patients were included if they met the following criteria: (1) meet the diagnostic criteria established by the Histiocyte Society in 2009; (2) relapsed or refractory (no response to first-line or second-line chemotherapy, or relapse after remission), or severe disease at initial diagnosis (Disease Activity Score ≥ 7 (Donadieu et al. 2004) and multiple organ involvement or risk organ involvement); 3)no previous treatment with BRAF^V600E^ inhibitors. Patients were excluded if they had not received vemurafenib for at least 4 weeks or were lost to follow-up.

The study adhered to the ethical standards outlined in the Declaration of Helsinki and received approval from the ethics committee of Sun Yat-sen Memorial Hospital. The legal guardian of each patient was informed about the benefits and drawbacks of treatments and provided informed consent before treatment commenced.

### Study design and procedures

The patients were treated with vemurafenib and chemotherapies according to the JLSG-02 protocol or CCHG-LCH-2019 protocol modified from LCH-III (Gadner et al. 2013; Morimoto et al. 2016). Vemurafenib was administered orally twice daily at a dose of 20 mg/kg for 1 year. The dose and duration of treatment were adjusted based on the patient’s tolerance and efficacy. Patients with severe disease at the initial diagnosis received vemurafenib in combination with CCHG-LCH-2019 protocol. The Initial Treatment One comprised weekly vincristine (1.5 mg/m^2^) and continuous oral prednisone (40 mg/m^2^/day for four weeks, tapering off over the subsequent 2 weeks) for a total of 6 weeks. Following Initial Treatment One, Initial Treatment Two comprised weekly vincristine (1.5 mg/m^2^) and 3 days of oral prednisone (40 mg/m^2^/day) every week for a total of 6 weeks. Maintenance treatment typically involved daily administration of 6-mercaptopurine (50 mg/m^2^/day), as well as pulses of prednisone (40 mg/m^2^/day for a period of 5 days) and vincristine (1.5 mg/m^2^) administered every 3 weeks. Patients who experienced relapse after remission, or who were refractory to prior chemotherapy, received vemurafenib in combination with Maintenance treatment of CCHG-LCH-2019 protocol or Induction B and Maintenance of the JLSG-02 protocol (Morimoto et al. 2016). The treatment details are outlined in S1.

As part of our study, we conducted routine examinations on all patients 1 week before treatment initiation, followed by subsequent follow-up evaluations every month thereafter. Patients underwent regular magnetic resonance imaging (MRI) or computed tomography (CT) scans every 6 months throughout their treatment to assess the efficacy of the therapy. Bone marrow biopsy, if applicable, was performed every 3 months during treatment until BRAF^V600E^ was no longer detectable. To detect BRAF^V600E^ mutations, we employed the amplification refractory mutation system polymerase chain reaction (ARMS PCR) method to analyze samples from lesions, bone marrow, and/or peripheral blood. BRAF^V600E^ mutation can be detected in as little as 1% of 10ngDNA samples by ARMS PCR.

### Criteria used to assess outcome

The disease status was evaluated based on the Histiocyte Society criteria, and the patients were classified as follows: (1) non-active disease (NAD): all signs or symptoms resolved, no evidence of disease; (2) active disease-better (AD-B): signs or symptoms regressed, no new lesion; (3) active disease-intermediate (AD-I): original signs or symptoms regressed, the new lesion appeared; (4) active disease-stable (AD-S): original signs or symptoms persist, no new lesions; (5) active disease-worse (AD-W): original signs or symptoms progressed, and/or new lesion appeared. Relapse was defined as the recurrence of disease activity after achieving AD-better and NAD. The last follow-up date was August 1, 2023. Progression-free survival (PFS) was defined as the percentage of patients who did not experience disease progression among all patients. Objective response rate (ORR) was defined as the percentage of patients who achieved AD-B and NAD among all patients. Relapse-free survival (RFS) was defined as the percentage of patients who did not experience relapse among those who achieved AD-B and NAD. Progress was defined as disease progression, relapse, secondary malignancy, or death. Adverse events were graded according to The Common Terminology Criteria for Adverse Events 5.0 and managed in compliance with relevant guidelines and regulations.

### Statistical analyses

Statistical analyses were conducted using SPSS software version 21.0. Continuous variables were presented as median (range) while count variables were provided as frequency or rate. For between-group comparisons of quantitative variables, the Mann–Whitney *U* test was utilized. Survival rates were estimated using the Kaplan–Meier method, and group comparisons were performed using the log-rank test. The threshold for statistical significance was set at 0.05.

## Result

### Patient

Seventeen patients, including seven males and ten females, received vemurafenib plus chemotherapy during the study period. No one received vemurafenib for less than 4 weeks or was lost to follow-up. The median age at diagnosis and initial treatment with vemurafenib was 2.8 years (range, 0.6–12.9 years) and 3.1 years (range, 0.6–12.9 years), respectively. Out of the total patients, 15 cases (88.2%) presented with multiple organ involvement, 11 cases (64.7%) had risk organ involvement (RO +), and 12 cases (70.6%) had CNS risk lesions (CNS-RISK +). Eleven patients failed to achieve remission after initial chemotherapy. Seven were classified as AD-I and four as AD-W, according to the HS criteria.

Before the initiation of MAPK inhibition, measurable BRAF^V600E^-mutated cells were detected in 14 patients, including 6 patients (6/12, 50.0%) with mutations in the bone marrow (BM), 12 patients (12/15, 80.0%) in focal tissue, and 2 patients (2/6, 33.3%) in peripheral blood (PB) (Table 1).

**Table 1:** Characteristics of the patients before vemurafenib treatment

PT:Sex, Age (Y)	RO	CNS-RISK	Affected organs	BRAF^V600E^		Previous treatment	Disease state	DSA before VMF
				Lesion	BM			
01: M, 4.1	–	+	Bone	+	–	Maintenance treatment	AD-I	3
02: M, 1.3	+	+	Bone, liver	+	–	Initial treatment	AD-I	6
03: F, 1.4	+	–	Skin, hematopoiesis, liver, spleen	+	+	None	–	11
04: M, 3.1	–	+	Bone, lung, CNS	+	–	Maintenance treatment	AD-W	5
05: M, 4.1	–	+	Bone	+	–	Maintenance treatment	AD-I	3
06: F, 1.0	+	+	Bone, liver, lung, hematopoiesis	NA	+	None	–	11
07: F, 8.5	–	+	Bone, lung	+	–	Maintenance treatment	AD-I	5
08: F, 2.8	–	+	Bone, skin, lymphaden	+	NA	Induction A of JLSG-02	AD-I	5
09: M, 1.3	+	+	Bone, skin, liver, spleen, hematopoiesis	+	+	Induction A & B of JLSG-02	AD-W	13
10: M, 3.6	+	–	Bone, spleen, hematopoiesis	+	–	Initial treatment	AD-I	5
11: F,12.9	+	+	Thyroid, hematopoiesis, CNS, liver, lung	–	NA	None	–	9
12: F, 1.2	+	–	Skin, liver, spleen, hematopoiesis, lung	+	NA	None	–	11
13: F, 3.4	–	+	Skin, CNS (diabetes insipidus)	–	NA	Initial treatment	AD-I	2
14: F, 1.0	+	–	Skin, liver, lung, hematopoiesis	–	NA	None	–	9
15: F, 3.1	+	–	Bone, lung, hematopoiesis	NA	+	Induction A of JLSG-02	AD-W	9
16: F, 0.7	+	+	Pituitary, shin, pericardium, lymphaden, hematopoiesis	+	+	Induction A of JLSG-02	AD-W	7
17: M, 0.6	+	+	Shin, lung, lymphaden, hematopoiesis	+	+	None	–	7

*RO* risk organ, *CNS* central nervous system, *BM* bone marrow, *AD-I* active disease-intermediate, *AD-W* active disease-worse, *DSA* Disease Activity Score, *VMF* vemurafenib

### Treatment

All patients received vemurafenib in combination with chemotherapy. Seven patients underwent treatment with vemurafenib combined with Initial and Maintenance treatment of CCHG-LCH-2019 protocol. Seven patients received vemurafenib combined with Maintenance treatment of CCHG-LCH-2019 protocol. Three patients received vemurafenib combined with Induction B and Maintenance of JLSG-02 protocol. The median dose of vemurafenib was 20 mg/kg.d (range, 12–30 mg/kg.d). Vemurafenib was administered to patients for a median treatment duration of 15 months (range, 1–38 months), and the median follow-up period was 32 months (range, 1–60 months) (Table 2).

**Table 2:** Response to treatment with vemurafenib plus chemotherapies

Parameters	
*Combined treatment*	
Initial and maintenance treatment, *N* (%)	7 (41.2%)
Maintenance treatment, *N* (%)	7 (41.2%)
Induction B and maintenance of JLSG-02, *N* (%)	3 (17.6%)
*Disease state*	
NAD, *N* (%)	9 (52.9%)
AD-B, *N* (%)Z	7 (41.2%)
AD-W, *N* (%)	1 (5.9%)
VMF withdraw, *N* (%)	15 (88.2%)
Withdrawal duration (M), media (Rang)	17 (3,30)
Reactivation, *N* (%)	4 (23.5%)
Follow-up duration (M), media (rang)	32 (1,60)

*NAD* non-active disease, *AD-B* active disease-better, *AD-W* active disease-worse, *VMF* vemurafenib

### Response

At the last visit, 16 of 17 patients achieved remission and the remission rate was 94.7%. One patient discontinued treatment after 1 month because of adverse events and was classified as AD-W. Molecular monitoring was conducted for six patients who had detectable BRAF^V600E^ mutations in the BM before treatment. Molecular remission was achieved in five patients, while one patient exhibited persistent detection of the bmBRAF^V600E^ mutation despite clinical symptom relief. At the last follow-up, this patient was still NAD on continued vemurafenib monotherapy. Chest CT scans were performed on seven patients with pulmonary involvement, and complete resolution of lesions was observed in two cases while the remaining five cases showed improvement. Fourteen patients achieved remission in 6 months (Table 3). Children treated with vemurafenib as primary treatment exhibited response after a median of 1 month (range, 1–3 months). Meanwhile, in children who received the combination therapy after failed initial chemotherapy, symptom improvements were observed after a median of 3 months (range, 1–14 months). The response time was shorter in the former group, although the difference is not statistically significant (*P* = 0.08).

**Table 3:** Disease state at each time piont after vemurafenib treatment (*N*, %)

	AD-B	AD-S	AD-I	AD-W	Discontinuation
1 month	6 (35.3)	9 (52.9)	1 (5.9)	1 (5.9)	0
2 months	6 (35.3)	8 (47.1)	1 (5.9)	0	2 (11.8)
3 months	10 (58.8)	5 (29.4)	0	0	2 (11.8)
6 months	13 (76.5)	1 (5.9)	0	0	3 (17.6)

*AD-B* active disease-better, *AD-S* active disease-stable, *AD-I* active disease-intermediate, *AD-W* active disease-worse

### Relapse and survival

At the last follow-up, 15 children discontinued vemurafenib after achieving AD-B or NAD. The cessation time ranged from 3 to 30 months, with a median of 17 months. Clinical reactivation occurred in three patients. We closely monitored the BRAF^V600E^ mutation of relapse patients in both BM and PB, while none of them converted to positive. One patient suffered molecular reactivation but continued clinical remission. Among these four relapse cases, two were treated with vemurafenib combined with maintenance therapy. One of them relapsed 17 months after stopping vemurafenib, manifesting as multiple new lesions in the skull, and was relieved after receiving chemotherapy with the cladribine and cytarabine regimen. The other relapsed 8 months after vemurafenib discontinuation. It manifested as multiple bone lesions and was relieved after vemurafenib monotherapy. The other two patients were treated with vemurafenib combined with CCHG-LCH-2019 protocol. One of them experienced relapse 3 months after vemurafenib withdrawal, manifesting as multiple new lesions in the skull, and the other had a molecular reactivation 19 months after stopping vemurafenib. Both of them responded with re-started vemurafenib monotherapy. All of them were NAD at the last follow-up (Table 2).

**Fig. 1:**
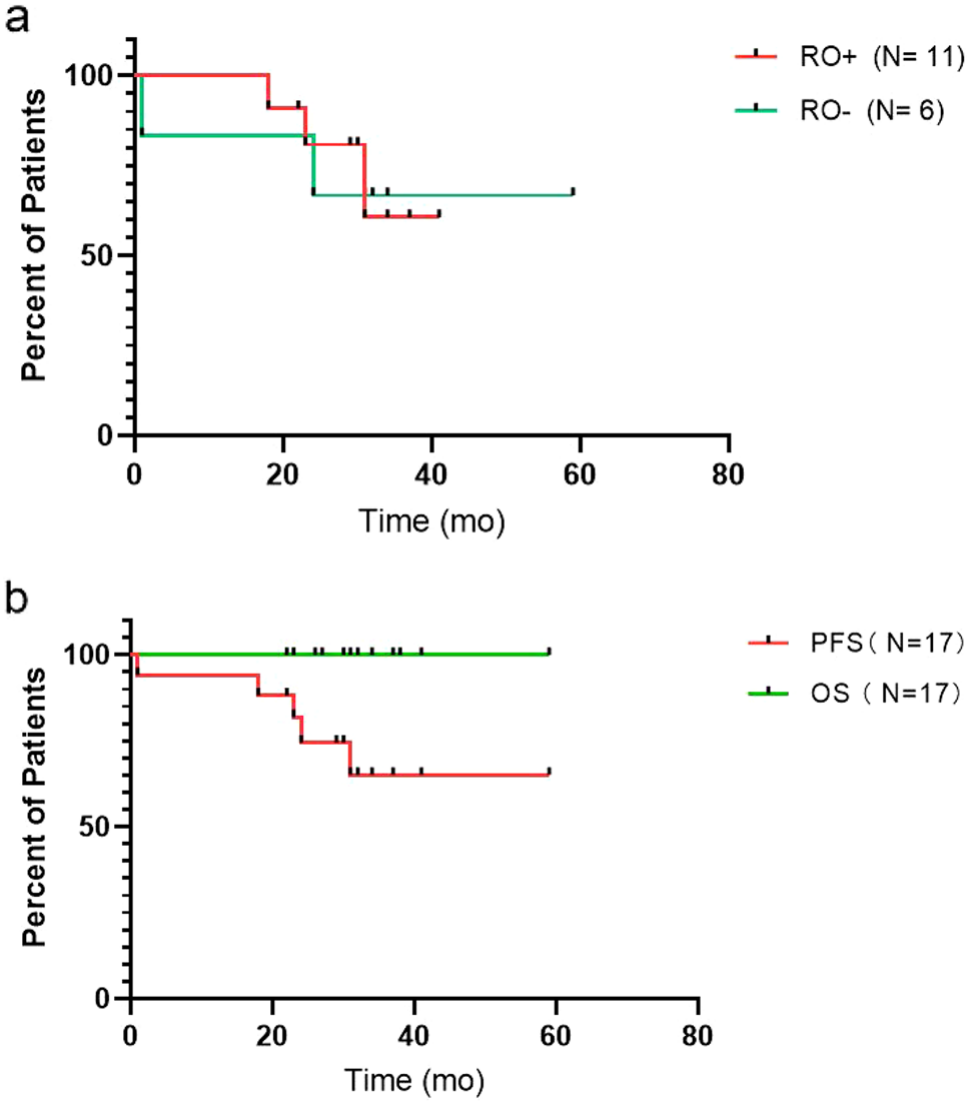
Survival curves. **a** Progression-free survival of the risk organ (RO) + group and RO–group. **b** Overall survival and progression-free survival of LCH patients treated with VFM

The relapse-free survival rate was 73.3% (11/15) and the median duration of remission (DOR) after cessation was 17 months. All patients survived, with a progression-free survival rate of 70.6% (12/17). No significant difference was observed in progression-free survival between patients in the RO + and RO- groups (Fig. 1).

### Adverse effects

Nine adverse events were observed in seven patients, including rash (five cases, 29.4%), pruritus (one case, 5.9%), arthritis (one case, 5.9%), cholangitis (one case, 5.9%), and liver dysfunction (one case, 5.9%). According to the Common Terminology Criteria for Adverse Events (CTCAE), five events were classified as grade 1 and four as grade 2. The majority of adverse events occurred in the early stages of treatment and were mostly resolved through treatment or observation alone. No hematological side effects or complications of grade 3 or higher were observed.

Discontinuation due to adverse events occurred in four patients (21.1%). Two of the patients were discontinued after 1 month. One of these two was discontinued due to grade 2 adverse events such as severe rashes, arthritis, and liver dysfunction, and the other was discontinued at the request of their parent due to repeated rashes. One patient was discontinued at 4 months due to rashes, and another at 7 months due to cholangitis. At the last follow-up, the former was NAD, while the latter experienced recurrence 17 months after discontinuation and achieved remission after receiving cladribine and cytarabine chemotherapy (Table 4).

**Table 4:** Adverse events in study population (*N*, %)

	Toxicity grade			Management	
	1	2	3	DR	TD
Skin rash	4 (23.5)	1 (5.9)	0	1 (5.9)	3 (17.6)
Cholangitis	0	1 (5.9)	0	0	1 (5.9)
Pruritus	1 (5.9)	0	0	0	0
Arthritis	0	1 (5.9)	0	0	1 (5.9)
Liver dysfunction	0	1 (5.9)	0	0	1 (5.9)

*DR* dose reduction, *TD* treatment discontinuation

## Discussion

LCH patients with BRAF^V600E^ mutations often have more severe disease presentations and are less likely to respond to first-line treatment (Heritier et al. 2016). In this study, we found that the combination of vemurafenib with hypotoxic conventional chemotherapies could rapidly induce a sustained and durable response in the majority of patients with severe or refractory LCH, and the associated side effects were tolerable. Our study showed that vemurafenib in combination with chemotherapy can achieve remission at the molecular level, which is difficult to achieve with vemurafenib monotherapy (Eckstein et al. 2019).

During the last decades, several treatment regimens have been demonstrated to be useful for severe LCH patients (Rigaud et al. 2016). However, all of them have their limitations. The combination of cladribine/Ara-C, as a salvage treatment, significantly improved the ORR and the survival rate of severe LCH patients, but the toxicity was high and the response was slow (Donadieu et al. 2015). As a single agent, the BRAF^V600E^ inhibitor can provide a rapid and robust clinical response, but the relapse rate after discontinuation is about 80% (Mohapatra et al. 2022; Wang et al. 2021). The clinical presentation of Langerhans cell histiocytosis (LCH) is influenced by the location of misdirected myeloid differentiation (Berres et al. 2014). Somatic BRAF^V600E^ mutations in hematopoietic precursors increase the risk of developing multiple organ involvement in LCH (Rodriguez-Galindo and Allen 2020; Poch et al. 2021). Moreover, the burden of these mutant clones correlates with the probability of relapse (Wang et al. 2021; Milne et al. 2023), emphasizing the importance of eliminating precursor cells for a cure. However, the persistence of BRAF^V600E^-positive mononuclear cells in both the blood and bone marrow, along with a high recurrence rate following drug withdrawal, suggests that vemurafenib monotherapy may be ineffective in completely eradicating LCH precursor cells (Eder et al. 2022; Donadieu et al. 2019). The cytotoxicity of chemotherapeutic agents can compensate for this deficiency. In our study, vemurafenib combined with cytotoxic chemotherapies was administrated in severe LCH patients. We observed a significantly high relapse-free rate of 73.3% (11/15) after discontinuation at a median follow-up of 34 months. About 83% (5/6) of patients achieved molecular remission in the bone marrow after treatment. Our results indicated that the combination of vemurafenib and chemotherapies was helpful in the complete elimination of BRAF^V600E^ mutant clones, thus leading to a more sustainable response.

LCH patients with BRAF^V600E^ mutations or those presenting with risk organ involvement or multiple organ involvement often exhibit a poor response to first-line treatments (Heritier et al. 2016). To enhance the efficacy of treatment in this patient group, we administered vemurafenib in combination with conventional chemotherapy to high-risk LCH patients following diagnosis. All patients demonstrated a positive response within 3 months and 66.6% of patients remained free of relapse at a median follow-up of 32 months. Vemurafenib as part of the initial chemotherapy regimen for patients with high-risk LCH may improve remission rates and reduce hospitalization costs for salvage therapy after relapse, but more data are needed to support this in the future.

The optimal combination strategy for vemurafenib therapy in Langerhans cell histiocytosis (LCH) patients remains uncertain, and there is limited data available on this therapeutic regimen. Dmitry-Evseev et al. administered a 6-month course of vemurafenib in combination with chemotherapy to young children or infants with aggressive multisystem LCH who were refractory to previous chemotherapy. Among the participants, four patients received vemurafenib combined with vinblastine and prednisolone, while five others received cytarabine and cladribine. However, only one patient who received vemurafenib plus cytarabine and cladribine successfully discontinued vemurafenib. It seems that a too brief course of vemurafenib may be insufficient for eradicating myeloid progenitors of LCH, even when combined with chemotherapy (Evseev et al. 2021). Evseev, D found that combined cytarabine (Ara-C) and 2'-chlorodeoxyadenosine (2-CdA) with vemurafenib achieved stable remission in LCH patients, but all patients experienced grade 3–4 hematological adverse reactions. One patient developed secondary myelodysplastic syndrome (sMDS) 14 months after vemurafenib therapy cessation (Evseev et al. 2023). In our study, we administered vemurafenib in combination with low-toxicity chemotherapy for over 12 months to the majority of patients, and most of them achieved clinical and molecular remission. These regimens were well-tolerated and suitable for administration in an outpatient setting, with a low incidence of adverse events (Tardieu et al. 2021). We also observed that three patients experienced relapse after drug withdrawal, one experienced molecular reactivation and one patient’s bmBRAF remained consistently positive, despite clinical symptoms having been alleviated. Whether an extended course of vemurafenib or more intensive chemotherapy regimens are needed to achieve a cure in these patients remains unknown. Although our study showed satisfactory efficacy of these regimens in most patients, further randomized trials are needed to confirm their efficacy in the treatment of LCH.

In conclusion, combination therapy of vemurafenib and chemotherapies can clear the bone marrow BRAFV600E gene load and maintain the response after drug withdrawal. As a first-line treatment for patients with high-risk Langerhans cell histiocytosis (LCH), vemurafenib combined with vinblastine and prednisone can potentially improve remission rates. Nevertheless, the optimal combination strategy remains unclear, and large-scale prospective studies are needed in the future to evaluate the effectiveness of the combination and to clarify the optimal regimen.

## Supplementary Information

Below is the link to the electronic supplementary material.

Supplementary file1 (DOCX 53 KB)

## Data Availability

The data presented in this study are available on request from the corresponding author. The data are not publicly available due to a privacy issue from the patients. No datasets were generated or analysed during the current study.
