# Supplementary figures and images for: Vemurafenib combined with chemotherapy achieved sustained remission in pediatric LCH: a multi-center observational study
Source: J Cancer Res Clin Oncol. 2024 Jan 17;150(1):12. doi: 10.1007/s00432-023-05551-y (PMC10794359; doi:10.1007/s00432-023-05551-y)

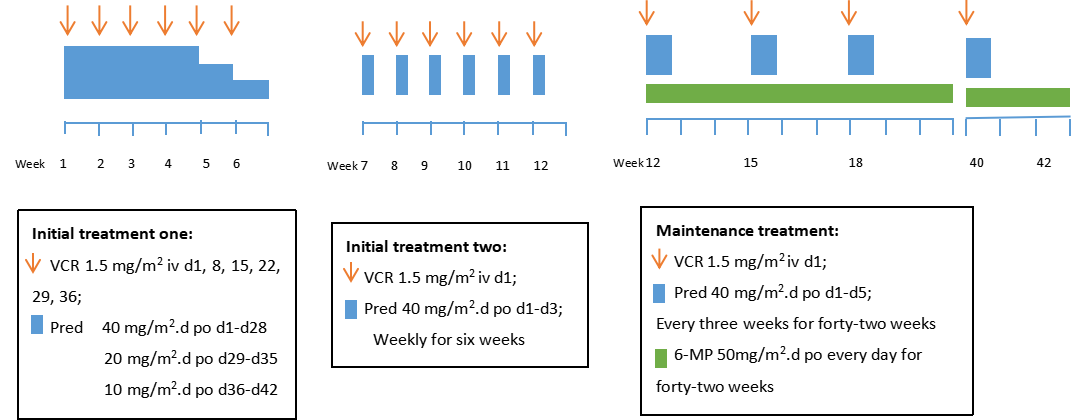


S1.Treatment protocol. *VCR* vincristine, *Pre* prednisolone, *6-MP* 6-mercap topurine.

Supplement: Supplementary file 1 — Supplementary file1 (DOCX 53 KB) [file 432_2023_5551_MOESM1_ESM.docx]
